# Monoaminergic tone supports conductance correlations and stabilizes activity features in pattern generating neurons of the lobster, *Panulirus interruptus*

**DOI:** 10.3389/fncir.2015.00063

**Published:** 2015-10-20

**Authors:** Wulf-Dieter Krenz, Anna R. Parker, Edmund Rodgers, Deborah J. Baro

**Affiliations:** Department of Biology, Georgia State UniversityAtlanta, GA, USA

**Keywords:** Kv4, HCN, homeostasis, plasticity, metaplasticity, conductance correlation, activity dependent, stomatogastric

## Abstract

Experimental and computational studies demonstrate that different sets of intrinsic and synaptic conductances can give rise to equivalent activity patterns. This is because the balance of conductances, not their absolute values, defines a given activity feature. Activity-dependent feedback mechanisms maintain neuronal conductance correlations and their corresponding activity features. This study demonstrates that tonic nM concentrations of monoamines enable slow, activity-dependent processes that can maintain a correlation between the transient potassium current (I_A_) and the hyperpolarization activated current (I_h_) over the long-term (i.e., regulatory change persists for hours after removal of modulator). Tonic 5 nM DA acted through an RNA interference silencing complex (RISC)- and RNA polymerase II-dependent mechanism to maintain a long-term positive correlation between I_A_ and I_h_ in the lateral pyloric neuron (LP) but not in the pyloric dilator neuron (PD). In contrast, tonic 5 nM 5HT maintained a RISC-dependent positive correlation between I_A_ and I_h_ in PD but not LP over the long-term. Tonic 5 nM OCT maintained a long-term negative correlation between I_A_ and I_h_ in PD but not LP; however, it was only revealed when RISC was inhibited. This study also demonstrated that monoaminergic tone can also preserve activity features over the long-term: the timing of LP activity, LP duty cycle and LP spike number per burst were maintained by tonic 5 nM DA. The data suggest that low-level monoaminergic tone acts through multiple slow processes to permit cell-specific, activity-dependent regulation of ionic conductances to maintain conductance correlations and their corresponding activity features over the long-term.

## Introduction

Most neurons have distinguishing activity features that identify them as belonging to a specific cell type. These fixed attributes are underpinned by conductance correlations that are maintained by activity-dependent feedback (Marder et al., 2015). Homeostatic mechanisms that preserve activity features are known to operate in a cell-specific (Fieblinger et al., 2014; Slomowitz et al., 2015) and age-dependent (Hennig et al., 2011; Mahoney et al., 2014) manner, but our understanding of how they maintain conductance correlations is limited. In particular, a given conductance correlation can be found in multiple neuronal cell types (Temporal et al., 2012). Is it maintained by the same or distinct mechanisms in each cell type? One cell type can exhibit several conductance correlations as well as correlations involving more than two conductances (Temporal et al., 2012; Zhao and Golowasch, 2012). Are all correlations in one cell type maintained by a centralized mechanism or distinct processes? It is not clear how tightly correlations are maintained or how they are shaped. This study uses a relatively simple experimental model to begin to answer some of these questions.

The lateral pyloric neuron (LP) is a pattern generating neuron that produces a continuous rhythmic output. The LP I_A_:I_h_ ratio (Temporal et al., 2012) and the timing of LP activity (Bucher et al., 2005; Goaillard et al., 2009) are maintained across individuals and lifetimes. Experimentally altering LP I_A_ initially disrupts the ratio and the timing of activity, but eventually, compensatory changes in LP I_h_ restore the ratio and timing (MacLean et al., 2003, 2005; Krenz et al., 2013, 2015). We discovered two mechanisms underpinning this compensation; each acted over a different time course, and both were enabled by tonic 5 nM DA.

The first mechanism was fast and acted over minutes (Krenz et al., 2013, 2015). Here the type 1 DA receptor (D1R)-PKA axis mediated two opposing effects: it increased LP I_h_ G_max_, and it permitted calcineurin to decrease LP I_h_ G_max_. Since calcineurin was activated by Ca^2+^-calmodulin, and Ca^2+^ concentration was defined by neuronal activity, tonic nM DA enabled rapid and reversible, bi-directional, activity-dependent regulation of LP I_h_ G_max_. This mechanism swiftly compensated for experimentally induced changes in I_A_; for example, reducing I_A_ with 4-AP altered neuronal activity, and in the presence, but not absence of tonic nM DA, the change in activity drove a compensatory reduction in I_h_ to restore the I_A_:I_h_ ratio and the timing of neuronal activity within minutes. We hypothesize that rapid, activity-dependent regulation of LP I_h_ G_max_ in nM DA normally compensates for activity-dependent changes in LP I_A_ *in vivo*. It is not clear if/how activity regulates LP I_A_ over the short-term, but studies on hippocampal neurons demonstrate rapid activity-dependent trafficking of the Kv4 (a.k.a. shal) channels mediating I_A_ (Kim et al., 2007). Theoretically, it is also possible for activity to influence the I_A_ window current resulting from Kv4 channel activation and inactivation kinetics and voltage dependencies.

Tonic nM DA also triggered a second, slow mechanism that did not produce immediate and reversible changes in ion currents; rather, peak effects occurred ~2–3 h after exposure to tonic nM DA and persisted for up to 1 day after removal of DA (Rodgers et al., 2011a,b, 2013; Krenz et al., 2014). This mechanism appeared to co-regulate LP I_A_ and I_h_ over the long-term; it produced a persistent, activity-independent increase in LP I_A_ G_max_ and permitted changes in activity to produce persistent adjustments to LP I_h_ G_max_. Like the fast mechanism, the slow mechanism was PKA-mediated, but in addition, RNA polymerase II that transcribes mRNA and microRNA, the RNA interference (RNAi) pathway that processes microRNA and mediates its effects, and mTOR-dependent translation were also required. Since the fast and slow tonic nM DA-enabled mechanisms are both mediated by PKA, and in both, DA permits activity to regulate LP I_h_ G_max_, we suspect both mechanisms belong to the same divergent D1R transduction cascade that functions to stabilize neuronal activity.

Unlike LP, the pattern generating pyloric dilator neuron (PD) expresses type 2 dopamine receptors (D2Rs) but not D1Rs (Oginsky et al., 2010). The PD I_A_:I_h_ ratio is also preserved across individuals (Temporal et al., 2012), and experimentally altering PD I_A_ elicits compensatory changes in PD I_h_ (MacLean et al., 2003). Nothing is known about the mechanism that preserves PD I_A_:I_h_, but the difference in receptor expression suggests it is distinct from the DA-enabled mechanism observed in LP. Here, we extend our studies to examine the persistent effects of three monoamines on LP and PD I_A_ and I_h_.

## Materials and Methods

### Animals

California spiny lobsters, *Panulirus interruptus*, were purchased from Marinus Scientific (Long Beach, CA, USA) and Catalina Offshore Products (San Diego, CA, USA). Lobsters were maintained at 16°C in aerated and filtered seawater. Animals were anesthetized on ice before dissection. There is no ethics committee approval required.

### Chemicals and Peptides

Tetrodotoxin (TTX) and flupenthixol were purchased from Tocris Bioscience (Bristol, UK), flavopiridol was from Selleckchem (Houston, TX, USA), and all other chemicals were purchased from Sigma-Aldrich (St. Louis, MI, USA). Peptides were synthesized by Biomatik (Wilmington, DE, USA). DA was made fresh every 30 min to minimize oxidation. Antagonists were administered 10 min before DA application. Concentrations of flavopiridol (100 nM) and 5,6-dichloro-1-β-D-ribobenzimidazole (DRB, 100 μM) were chosen based on previously demonstrated effective dosages (Chao and Price, 2001; Bensaude, 2011; Yuan and Burrell, 2013; Krenz et al., 2014).

### Experimental Preparation

**Figure 1:**
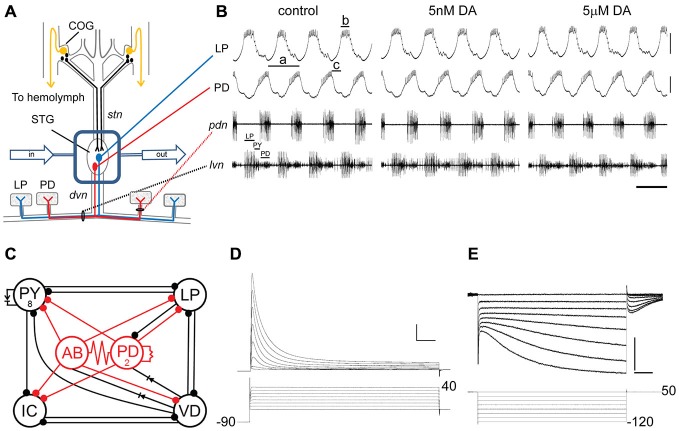
**The experimental model. (A)** *In situ* preparation. The stomatogastric nervous system (STNS) is dissected and pinned in a dish. The commissural ganglia (CoGs) contain DA neurons that project to the stomatogastric ganglion (STG; black) and L-cells, which are the source of neurohormonal DA (gold). The well surrounding the STG (blue rectangle) is continuously superfused with saline (in/out arrows). There are ~30 neurons in the STG; two are drawn: pyloric dilator (PD), lateral pyloric (LP). Network neurons interact locally within the STG neuropil and can project axons to striated muscles surrounding the foregut. The diagram shows that PD and LP neurons project their axons through identified nerves to innervate muscles (rectangles). **(B)** Spontaneous pyloric network output. The top two traces are intra-cellular recordings from the *in situ* preparation diagrammed in **(A)**. Lowercase letters indicate measured parameters: a, period; b, LP burst duration; c, LP-on delay. The bottom two traces represent extra-cellular recordings from identified motor nerves containing pyloric neuron axons. The spikes from three pyloric neurons are indicated on the lateral ventricular nerve (*lvn*). These simultaneous recordings demonstrate the spontaneous, recurrent, rhythmic motor pattern produced by the circuit; scale bars, 10 mV and 500 ms. **(C)** The pyloric circuit. The pyloric network comprises 14 neurons. The diagram represents pyloric neuron interactions within the STG. Open circles represent the six cell types, numbers indicate more than one cell within a cell type: anterior burster (AB), inferior cardiac (IC), ventricular dilator (VD); pyloric constrictor (PY); filled circles, inhibitory chemical synapses; resistors and diodes, electrical coupling; red, pacemaker kernel and its output connections. **(D,E)** Typical two electrode voltage clamp measure of I_A_ **(D)** and I_h_ **(E)**. Top: current traces; Bottom: voltage protocol; I_A_ scale bars, 50 ms and 100 nA; I_h_ scale bars, 500 ms and 5 nA.

The stomatogastric nervous system (STNS) experimental preparation is shown in Figure 1A. The STNS was dissected and pinned in a Sylgard lined Petri dish using standard techniques (Selverston et al., 1976). The stomatogastric ganglion (STG) was desheathed and isolated with a Vaseline well. The STG was superfused with saline consisting of (in mM) 479 NaCl, 12.8 KCl, 13.7 CaCl_2_, 39 Na_2_SO_4_, 10 MgSO_4_, 2 glucose, 4.99 HEPES, 5 TES at pH 7.4. Extracellular recordings from the pyloric dilator nerve (*pdn*) and lateral ventricular nerve (*lvn*) were obtained with stainless steel pin electrodes and a differential AC amplifier (A-M Systems, Everett, WA, USA). Intracellular somatic recordings were obtained using glass microelectrodes filled with 3M KCl (20–30 MΩ) and Axoclamp 2B or 900A amplifiers (Axon Instruments, Foster City, CA, USA). Neurons were identified by correlating action potentials from somatic intracellular recordings with extracellularly recorded action potentials on identified motor nerves, and by their characteristic shape and timing of oscillations. The process of dissection and cell identification took ~3–5 h. All experiments were done at 19–22°C as measured with a probe in the bath. Temperature did not change by more than 1°C during an experiment.

### Motor Pattern Analyses

Pyloric activity on extracellular electrodes was recorded with Axoscope v8.2 software (Molecular Devices). Extracellular recordings were analyzed using DataView v6.3.2 (Heitler, 2009) to determine cycle period and frequency, spikes per burst, burst duration, duty cycle, intraburst spike frequency, LP-on delay, and LP phase. Reported values for all parameters represent a 10 cycle average. One cycle period was defined as the last spike in one PD burst to the last spike in the next PD burst (Figure 1Ba). The LP-on phase, which is the point in the cycle where LP begin firings, was measured as the time between the last spike in PD and the first spike in LP (termed LP-on delay; Figure 1Bc) divided by cycle period. LP duty cycle was calculated as LP burst duration (Figure 1Bb) divided by cycle period. Intraburst spike frequency was calculated as LP spikes per burst divided by LP burst duration.

### Somatic Two-Electrode Voltage Clamp (TEVC)

For TEVC of I_h_, the well surrounding the STG was superfused for 5 min with blocking saline: saline containing 10^−6^ M picrotoxin to block inhibitory glutamatergic synaptic inputs, 10^−7^ M TTX to block voltage-gated Na^+^ channels, 2 × 10^−2^ M tetraethylammonium (TEA) to block voltage-gated K^+^ channels, 2 × 10^−4^ M cadmium chloride (CdCl_2_) to block Ca^2+^-and Ca^2+^-dependent channels. Neurons were impaled with two micropipettes (8–10 MΩ when filled with 3 M KCl) connected to Axoclamp 2B or 900A amplifiers (Molecular Devices, Foster City, CA, USA). Neurons were clamped to a −50 mV holding potential using pClamp software. I_h_ was elicited using a series of 4 s hyperpolarizing voltage steps, from −60 to −120 mV in 10 mV increments with 6 s between steps. Steady state peak currents were measured by fitting the current trace back to the beginning of the hyperpolarizing voltage step or by subtracting the initial fast leak current from the slowly developing peak of I_h_ at the end of each negative voltage step. Currents were converted to conductance (G = I_peak_/(V_m_−V_rev_)) and fitted to Boltzmann equations, assuming V_rev_ I_h_ = −35 mV (Kiehn and Harris-Warrick, 1992). I_A_ was measured using a digital subtraction method as previously described in Baro et al. (1997). Two sets of current traces were obtained: in the first series, the command potential was stepped from −50 to −90 mV for 200 ms to remove resting inactivation. The deinactivating prepulse was immediately followed by a 400 ms testpulse to activate the channels. Activation pulses ranged from −40 to +40 mV in 10 mV increments. In the second series, the hyperpolarizing prepulse was set to −40 mV to inactivate I_A_. The two sets of traces were digitally subtracted to obtain I_A_. Currents were converted to conductance (G = I_peak_/(V_m_−V_rev_)) and fitted to 1st order Boltzmann equations, assuming V_rev_ I_A_ = −86 mV (Eisen and Marder, 1982). TEVC was done at 19–22°C as measured with a probe in the bath. Temperature did not change by more than 1°C during any given experiment. We attempted to measure I_A_ and I_h_ in LP and in both PD neurons in every experiment, but we were often unsuccessful because all three neurons were not identifiable in every preparation and/or technical difficulties prevent good voltage clamp recordings of both currents in all cells.

### Ago Knock Down

Argonaute (Ago) is a protein component of the RNA interference silencing complex (RISC). RISC functions in the RNAi pathway that processes and mediates the effects of double-stranded RNAs, such as microRNAs. In these studies we disrupt the RNAi pathway by blocking Ago function. A 314 base pair fragment representing nucleotides 1365–1679 from the *Panulirus* Ago cDNA (Accession number KF602070) was cloned into the pDrive vector (Qiagen) using the instructions supplied by the manufacturer. The Ago fragment was flanked by T7 and SP6 promoters. The cloned fragment served as a template in T7 and SP6 *in vitro* transcription reactions using MegaScript RNAi Kits (Ambion) and instructions provided by the manufacturer. The resulting complementary transcripts were mixed, heated to 70°C for 5 min and annealed at room temperature overnight to produce double-stranded (ds)Ago RNA. As a control, a dsGFP RNA was also generated. An EGFP-containing plasmid (Clontech) served as a template in a PCR with specific GFP primers (Forward: CAAGGGCGAGGAGCTGTTCA, Reverse: GGTGTCGCCCTCGAACTTCA). The resulting 349 base pair fragment was then cloned into pDrive; the clone served as a template for T7 and SP6 *in vitro* transcription reactions; and the resulting RNA was annealed. The dsRNA was suspended in sterile filtered 0.3M KCl and iontophoresed (−10 nA for 10 min) into identified neurons. The preparation was then incubated overnight at room temperature in L-15 media (MacLean et al., 2003). The following day the media was removed and replaced with saline prior to performing experiments.

### Single Cell PCR

Electrophysiologically identified PD cells were removed from the STG: the ganglion was incubated with 1.2 mg/ml of collagenase type IA (Sigma-Aldrich, St. Louis, MO, USA) until the cells were amenable to extraction with a fire-polished microelectrode. Cells were immediately placed on dry ice and stored at −80°C until reverse transcription. PD cells were processed for RT-PCR by using a modified cells-to cDNA kit protocol (Ambion, Austin, TX, USA). First, 9 μl of lysis buffer was added to the cell and incubated at 75°C for 10 min. Next, 0.2 ml of DNase1 was added to lysis buffer and incubated for 15 min at 37°C, and then again at 75°C for an additional 5 min for inactivation. RNA was then reverse transcribed as per the manufacturer’s instructions. The resulting first-strand cDNA for a given cell was then aliquotted into two tubes, each containing a primer set for either Ago (experimental, should change with treatment) or α-tubulin (control, should not change with treatment). Then 2 μl from the reverse transcription reaction was added to 23 μl of PCR mix containing Advantage Taq polymerase (ClonTech, Palo Alto, CA, USA), and used according to the manufacturer’s instructions. All reactions for one STG were run simultaneously under the following PCR conditions: 95°C for 1 min, 60°C for 1 min, 68°C for 45 s, for 35 cycles. PCR products were run on a polyacrylamide gel and visualized with ethidium bromide. We chose a region of AGO for the Single cell PCR that was not in the region targeted with the dsRNA. The forward and reverse primers used were as follows:

Ago forward: CACCAGTCTATGCGGAAGTGAAG
Ago reverse: GGTCTGATGCCTGGGACTAGAAT
α-tubulin forward: 5-GACTACGGCAAGAAGAGCAAACT-3
α-tubulin reverse: 5-TGTTCATGTTCTGCGGCAGATGTC-3.

### Peptide Injection

Injection of an Ago hook peptide was used to acutely disrupt LP RISC and microRNA function. The his-tagged hook (HHHHHHPDNGTSAWGEPNESSPGWGEMD) and mutant hook (HHHHHHPDNGTSvavEPNESSPvavEMD) peptides were diluted in water to a working concentration of 10 ng/ml and fast green was added to 0.04% to visualize injections. Microloaders (Eppendorf) were used to directly fill glass pipettes (8–15 MΩ when filled with 3M KCL) with the solution (i.e., no backfilling). Because of the high resistance of the peptide solution, pipette tips were broken before injection by gently touching a Kim wipe. The peptide was pressure injected into neurons using a Picospritzer III (General Valve/Parker Hannifin). Only two pressure pulses (on average 32 psi and 47 ms) separated by 30 s were applied. The preparation was then superfused with saline for 1 h prior to performing an experiment. Intracellular recording during the injection showed that the injection had no effect on neuronal voltage envelopes and firing properties. Extracellular recordings were used to continuously monitor neuronal activity before during and for 1 h after peptide injection and no change was observed.

### Statistical Analyses

The data were checked for normality using D’Agostino-Pearson omnibus, Shapiro-Wilk or Kolmogorov-Smirnov normality tests, in that order depending upon the n. Normal data were analyzed using parametric statistics, and non-parametric tests were used for the one dataset that was not normally distributed (PD I_h_ in 5 nM DA). The Prism Statistical software package (Graphpad) was used for statistical analyses including correlations, *t*-tests, one-way ANOVAs and Kruskal-Wallis comparisons. Significance threshold was set at *p* < 0.05 in all cases. Statistical outliers were excluded if the values fell greater than 2 standard deviations from the mean. Means and standard errors are presented unless otherwise noted. ANOVAs were followed by either Dunnett’s *post hoc* tests that compare all columns to a single column or Tukey’s *post hoc* tests that make all pairwise comparisons.

## Results

### Persistent Effects of Monoamines on LP

Our experimental model is the pyloric network in the crustacean STNS (Marder and Bucher, 2007) of the spiny lobster, *Panulirus interruptus* (Figure 1A). The 14-neuron pyloric pattern generator produces a spontaneous rhythmic output *in situ* that can be recorded with intracellular and extracellular electrodes (Figure 1B). The network neurons, including LP and PD, are located in the STG where they interact with one another through electrical and chemical couplings (Figure 1C). These neurons also send an axon out of the ganglion through identified motor nerves to innervate identified muscles. This study focuses on the single LP and the two PD neurons.

The STG receives DA, OCT and 5HT transmissions. Projection neurons in the commissural ganglia (COGs) release DA through volume transmission (Oginsky et al., 2010), resulting in transient increases in DA (~μM) near release sites, as well as tonic nM levels of DA (Zoli et al., 1998). The firing properties of both LP and PD are immediately and reversibly altered in response to bath applied μM DA (Flamm and Harris-Warrick, 1986a,b) but not nM DA (Figure 1B). LP exclusively expresses D1Rs and PD exclusively expresses D2Rs (Oginsky et al., 2010; Zhang et al., 2010). Both receptor types act through canonical cascades in the STG (Clark et al., 2008) and behave like their mammalian counterparts when expressed in human embryonic tissue culture cells (Clark and Baro, 2006, 2007). Octopaminergic modulatory neurons are also thought to reside in the COGs (Barker et al., 1979). Both LP and PD immediately and reversibly respond to bath applied μM OCT (Flamm and Harris-Warrick, 1986a,b). Superfusion of nM OCT did not appear to alter activity. OCT receptor distributions are unknown. In *P. interruptus*, 5HT is not released by modulatory neurons; it is a neurohormone that is expressed at detectable levels in the hemolymph bathing the STG (Beltz, 1999). Bath application of μM 5HT produced an immediate and reversible response in LP but not PD (Flamm and Harris-Warrick, 1986a,b). Superfusion of nM 5HT did not appear to alter activity. PD neurons express 5HT2_βPan_ receptors; it is not known whether they express additional 5HT receptors (Clark et al., 2004). 5HT receptor expression patterns are not known for LP.

Our overarching hypothesis is that phasic modulatory release (~μM) produces immediate and reversible alterations in neuronal and circuit output to fit the situation at hand, while tonic nM levels of modulators stabilize specific activity features by maintaining their underlying conductance correlations. The persistent effects of low-level tonic monoamines are studied here by continuously superfusing the STG *in situ* in order to remove neurohormones and prevent the accumulation of tonic modulators due to volume transmission (Figure 1A). We expect that phasic transmissions may not be greatly disrupted by this experimental design. After neurons were electrophysiologically identified *in situ* during constant saline superfusion, a given modulator was added to the saline superfusate at a concentration of 5 nM for 1 h. This was followed by a 2 h superfusion with saline alone, as we previously demonstrated that this was when the persistent, DA-induced peak effects occur for both I_A_ (Rodgers et al., 2013) and I_h_ (Krenz et al., 2014). At the end of the 2 h wash, the preparation was superfused for 5 min with saline containing inhibitors that block non-I_A_ and -I_h_ intrinsic and synaptic conductances (see “Materials and Methods” Section), after which, I_A_ and I_h_ were measured with TEVC in the same blocking saline (Figures 1D,E). This experiment (with slight variations) was performed throughout the study presented here.

**Figure 2:**
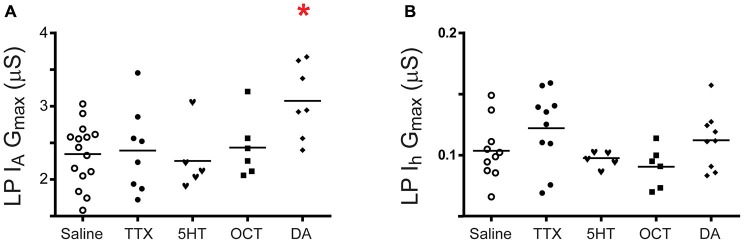
**DA, but not 5HT, OCT or a change in activity produce LP I_A_ plasticity, and I_h_ plasticity is not observed under any condition.** Preparations were superfused with saline, 5 nM of a given monoamine or 100 nM tetrodotoxin (TTX) for 1 h followed by a 2 h superfusion with saline and two-electrode voltage clamp (TEVC) measures of LP I_A_ **(A)** and LP I_h_ **(B).** G_max_ are plotted; each symbol is one preparation; the lines represent means. Data were analyzed with one-way ANOVAs followed by Dunnett’s *post hoc* tests that compared monoamine and TTX treatment groups to the saline treatment group. Asterisks indicate significant differences between monoamine and saline treatment groups. LP I_A_: *F*_(4,36)_ = 3.378, *p* = 0.0191; LP I_h_: *F*_(4,35)_ = 2.060, *p* = 0.1072.

**Figure 3:**
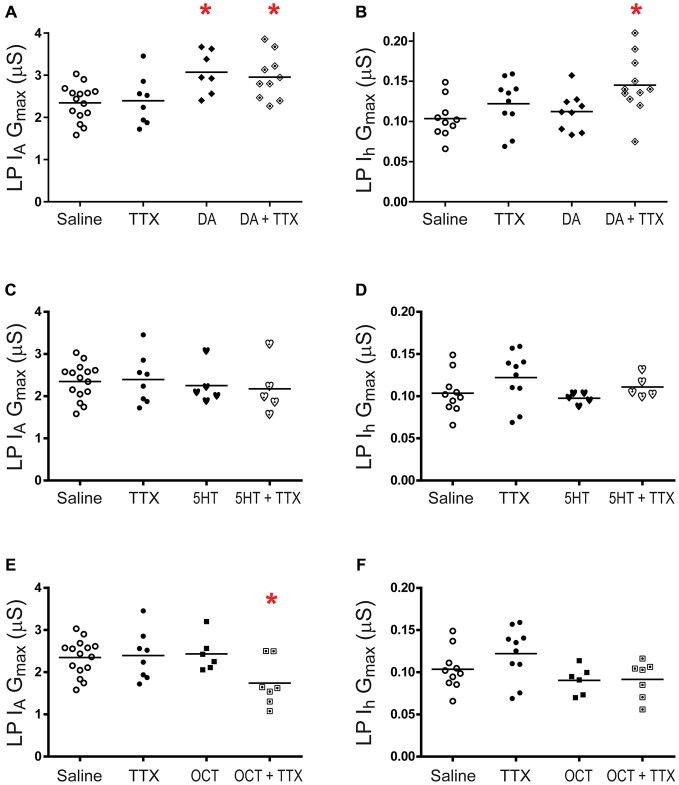
**LP metaplasticity.** Preparations were superfused with 5 nM of a given monoamine + 100 nM TTX for 1 h followed by a 2 h superfusion with saline and TEVC measures of LP I_A_ and I_h_. G_max_ are plotted; each symbol is one preparation; the lines represent means. One way ANOVAs with Dunnett’s *post hoc* tests compared monoamine alone, TTX alone and monoamine + TTX treatment groups to the saline control. Asterisks indicate significantly different from control. **(A)** LP I_A_ in DA, *F*_(3,36)_ = 5.533, *p* = 0.0031;** (B)** LP I_h_ in DA, *F*_(3,36)_ = 3.844, *p* = 0.0174; **(C)** LP I_A_ in 5HT, *F*_(3,29)_ = 0.2404, *p* = 0.8674; **(D)** LP I_h_ in 5HT *F*_(3,26)_ = 0.1538, *p* = 0.2282; **(E)** LP I_A_ in OCT, *F*_(3,32)_ = 3.263, *p* = 0.0340; **(F)** LP I_h_ in OCT, *F*_(3,29)_ = 2.869, *p* = 0.0536.

Previous work suggested that plasticity and metaplasticity were involved in long-term co-regulation of LP I_A_ and I_h_ in 5 nM DA (Rodgers et al., 2011a,b, 2013; Krenz et al., 2014). Here, we broadly define plasticity as a stimulus-induced change that significantly outlasts the stimulus (Mitchell and Johnson, 2003). According to this definition, monoamines and neuronal activity are both capable of producing plasticity. Metaplasticity occurs when one modulator enables a second modulator, or changes its modulatory effect (Fields and Mitchell, 2015). The first set of experiments investigated LP plasticity by determining whether a given monoamine, or a TTX activity blockade alone, produced a significant persistent change in I_A_ G_max_ or I_h_ G_max_ relative to saline controls. DA is shown for completeness. It should be noted that TTX application will also reversibly block spike-evoked modulatory release to the STG (i.e., phasic transmission). The data indicated that only tonic nM DA could elicit I_A_ plasticity (Figure 2). The next set of experiments investigated I_A_ and I_h_ metaplasticity (Figure 3). A given monoamine was co-applied with TTX for 1 h followed by a 2 h saline superfusion and TEVC to measure LP I_A_ and I_h_. G_max_ for the monoamine alone, TTX alone and TTX + monoamine treatment groups were compared to the G_max_ for the saline treatment group. As previously described (Rodgers et al., 2011a; Krenz et al., 2014), LP I_h_ metaplasticity was observed in 5 nM DA (Figure 3B); that is to say, neither DA nor TTX alone modulated LP I_h_ in a statistically significant manner, but when co-applied, they produced a significant, persistent change in LP I_h_. In addition, we observed LP I_A_ metaplasticity in 5 nM OCT + TTX (Figure 3E). The voltage dependence of I_A_ and I_h_ activation and inactivation were not persistently altered in any of these experiments.

### Tonic nM DA Acts Through a RISC- and RNA Polymerase II-Mediated Mechanism to Preserve the LP I_A_:I_h_ Ratio and Several Activity Features over the Long-Term

**Figure 4:**
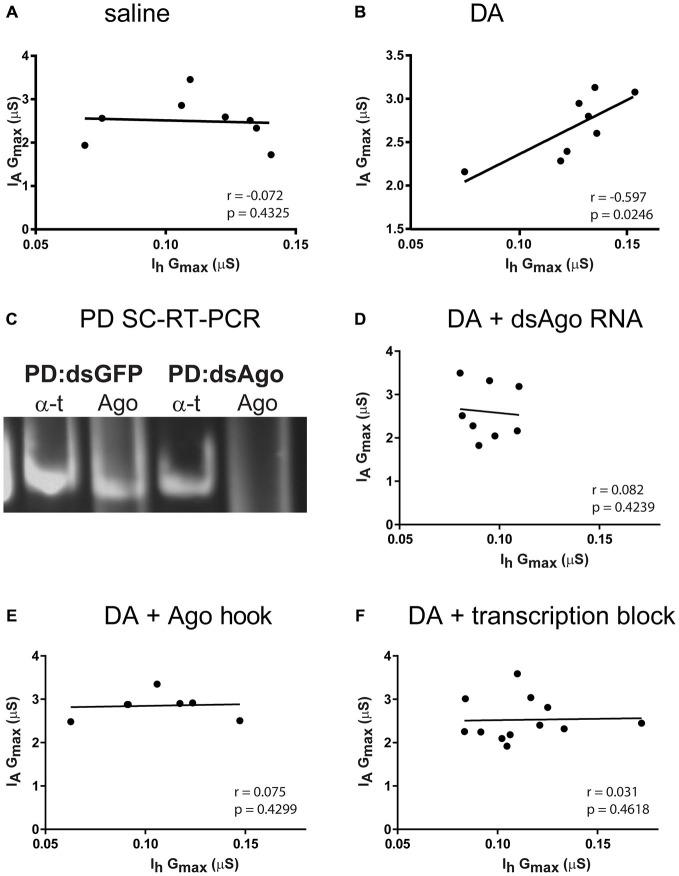
**Tonic nM DA acts through a RNA interference silencing complex (RISC)- and RNA Polymerase II-mediated mechanism to preserve the LP I_A_:I_h_ ratio over the long-term. (A,B)** LP I_A_ and I_h_ are correlated over the long-term in DA but not saline. Preparations were superfused with saline + TTX **(A)** or 5 nM DA + TTX **(B)** for 1 h, followed by a 2 h saline superfusion and TEVC to measure LP I_A_ and I_h_. Maximal conductances (G_max_) were plotted for each individual preparation (filled circles), and the data in the scatterplots were used to compute the Pearson correlation coefficient (*r*) and associated *p*-value that are recorded on each plot. In addition, lines of best fit were drawn on the scatterplots. The slope of the best fit line is also indicated for correlated conductances. **(C)** Injection of dsAgo RNA abolished Ago transcripts within 24 h. The two PD neurons were electrophysiologically identified in one STG preparation. One PD neuron was injected with dsGFP RNA (control) and the other was injected with dsAgo RNA. The next day the two PD neurons were physically isolated and each served as a template in a single cell-reverse transcription reaction (SC-RT). A given SC-RT product then served as the template in each of two PCRs using alpha tubulin (αT) and Ago primers in separate reactions. PCR products were analyzed with gel electrophoresis and ethidium bromide staining. A representative result is shown (*n* = 3 STG preparations). **(D,E)** RISC is necessary to maintain the correlation between LP I_A_ and I_h_ in DA. In **(D)** the LP neuron from a given preparation was identified and injected with dsAgo RNA. The following day the preparation was superfused with 5 nM DA + TTX for 1 h, followed by a 2 h saline superfusion and TEVC to measure LP I_A_ and I_h_. The scatterplot shows data for each individual (filled circles). In **(E)** LP neurons were injected with the Ago hook peptide to prevent RISC formation and superfused with saline for 1 h. Preparations were then superfused with 5 nM DA + TTX for 1 h followed by a 2 h saline superfusion and TEVC to measure LP I_A_ and I_h_. The scatterplot shows data for each individual (filled circles). **(F)** RNA Polymerase II transcription is necessary to maintain the correlation between LP I_A_ and I_h_ in DA. Preparations were superfused with a transcription blocker for 10 min, followed by a 1 h superfusion with the same transcription blocker + 5 nM DA + TTX, followed by a 2 h superfusion with saline and TEVC to measure LP I_A_ and I_h_. The scatterplot shows data for each individual (filled circles).

**Figure 5:**
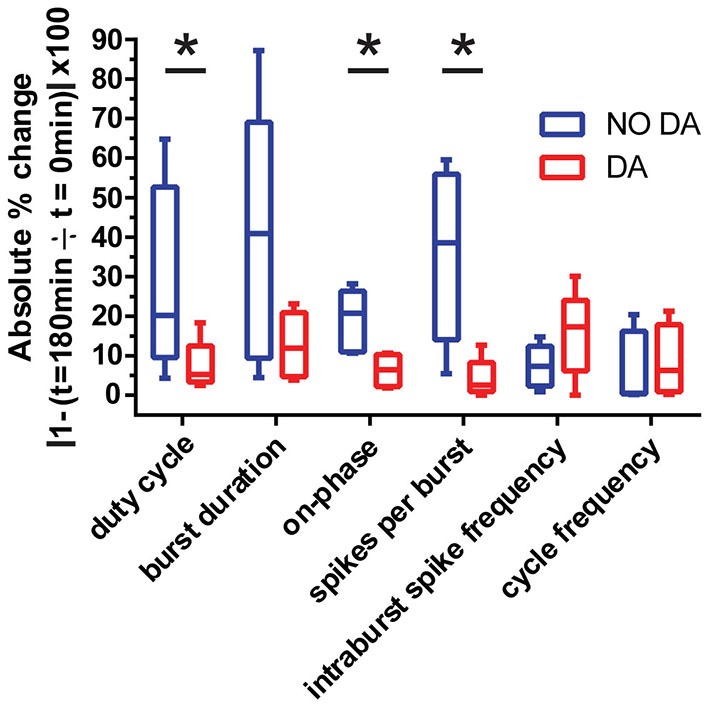
**Maintenance of the LP I_A_:I_h_ ratio preserves LP duty cycle, LP-on phase and LP spike number per burst.** At *t* = 0 LP received a continuous 1 h hyperpolarizing current injection to reduce burst duration by 25% while being superfused with either saline (blue) or 5 nM DA (red). Preparations were then superfused with saline for 2 h. Extracellular recordings were maintained throughout the experiment and used to measure LP duty cycle (burst duration ÷ cycle period, b ÷ a in Figure 1B), burst duration, LP-on phase (LP-on delay ÷ cycle period, c ÷ a in Figure 1B), spikes per burst, intraburst spike frequency and cycle frequency at *t* = 0 and *t* = 180 min, *n* ≥ 5 per treatment group. Tukey box plots illustrate the absolute percentage changes over time for each treatment group (∣1 − (*t* = 180 ÷ *t* = 0)∣ × 100). Absolute values were chosen instead of averages because some parameters could increase or decrease over time depending upon the preparation, and averaging did not reflect the degree of variability observed. The two treatment groups were compared with unpaired *t*-tests. Asterisks indicate significant differences: Duty cycle: *p* = 0.043853, Burst duration: *p* = 0.056097, LP-on phase: *p* = 0.011911, Spikes per burst; *p* = 0.007098, Intraburst spike frequency: *p* = 0.08439, Cycle frequency: *p* = 0.365394.

Our hypothesis predicts that tonic nM DA can maintain LP conductance correlations. The data suggested that if DA co-regulated LP I_A_ and I_h_ to maintain a conductance correlation, then the exact correlation would depend upon LP activity (see Figure 3). We therefore examined the correlation between I_A_ and I_h_ in individual LP neurons showing the same activity level (Figure 4). After dissection and cell identification, preparations were superfused for 1 h with saline containing TTX (Figure 4A) or 5 nM DA + TTX (Figure 4B). After an additional 2 h saline superfusion, LP I_A_ and I_h_ were measured with TEVC. I_A_ G_max_ and I_h_ G_max_ were plotted for each individual and the data were analyzed for correlations. A positive correlation between LP I_A_ G_max_ and I_h_ G_max_ was observed in the 5 nM DA (Figure 4B) but not in the saline (Figure 4A) treatment group.

We previously demonstrated that 5 nM DA acted through Polymerase II transcription, RNAi and mTOR-dependent translation to produce long-term regulation of LP I_A_ and I_h_ (Krenz et al., 2014). We wished to determine if these components were also necessary to maintain the positive correlation between LP I_A_ and I_h_. In the next three sets of experiments, we determined the effect of inhibiting RNAi or transcription on DA-enabled long-term maintenance of the LP I_A_:I_h_ ratio under conditions of complete activity blockade, i.e., 5 nM DA + TTX.

In the first two sets of experiments, the RNAi pathway was inhibited by disrupting one of its key components, the RISC. Among other things, RISC comprises an Argonaut (Ago) and TNRC6 heterodimer that is essential for RISC formation and function (Till et al., 2007). Knocking out either protein, or preventing their interaction, inhibits the RNAi pathway. In the first set of experiments double stranded (ds)RNA was used to knock out Ago expression: when dsAgo RNA was injected into a pyloric neuron and the ganglion was incubated for 24 h, Ago RNA was undetectable in the injected cell (Figure 4C). LP neurons were injected with dsAgo RNA and incubated overnight. The next day DA + TTX was bath applied for 1 h, washed out for 2 h and LP I_A_ and I_h_ were measured with TEVC. Under these conditions, there was no positive correlation between LP I_A_ and I_h_ (Figure 4D). In the second set of experiments, injection of an Ago hook was used to acutely disrupt the dimerization of Ago and TNRC6 as previously described in Krenz et al. (2014). Briefly, a small peptide that represents the Ago binding domain on the TNRC6 protein, termed the Ago hook, out-competes TNRC6 for binding to Ago and prevents dimerization and RISC formation. LP was injected with the Ago hook peptide and superfused with saline for 1 h; the preparation was then superfused with 5 nM DA + TTX for 1 h followed by a 2 h saline superfusion and TEVC measurements of I_A_ and I_h_. Under these conditions, there was no long-term correlation between LP I_A_ and I_h_ (Figure 4E). Together these data suggested that RISC was necessary for the maintenance of the LP I_A_:I_h_ ratio over the long-term in tonic nM DA.

A third set of experiments tested whether or not RNA Polymerase II transcription was necessary for DA-enabled long-term maintenance of the LP I_A_:I_h_ ratio using three transcription blockers: Flavopiridol and DRB block Polymerase II elongation and actinomycin D intercalates into the DNA to block all transcription (Bensaude, 2011). Preparations were superfused for 1 h with one of the three transcription blockers + 5 nM DA + TTX followed by a 2 h washout and TEVC measures of LP I_A_ and I_h_. Under these conditions, the positive correlation between LP I_A_ and I_h_ was not observed (Figure 4F), suggesting that Polymerase II transcription was necessary to maintain the correlation. In sum, tonic nM DA enabled long-term maintenance of the LP I_A_:I_h_ ratio, and transcription and a functional RNAi pathway were necessary for this to occur.

Conductance correlations underpin specific activity features (MacLean et al., 2003, 2005; Burdakov, 2005; Barnett et al., 2010; Chen et al., 2010; Hudson and Prinz, 2010; Soofi et al., 2012; Zhao and Golowasch, 2012; Bonin et al., 2013; Franci et al., 2013; Krenz et al., 2013, 2015; Lamb and Calabrese, 2013). If tonic 5 nM DA maintains conductance correlations, then it should also preserve their associated activity features. We next examined which activity features were stabilized by the persistent effects of 5 nM DA. At *t* = 0 the STG was superfused with saline or 5 nM DA for 1 h. At the same time, hyperpolarizing current was injected into the LP to reduce burst duration by 25%. After this 1 h treatment, preparations were superfused with saline from *t* = 60 min to *t* = 180 min, *n* = 5 per treatment group. LP activity was recorded with extracellular electrodes throughout the experiment (Figure 1B). These recordings were used to measure spontaneous LP activity at *t* = 0 and *t* = 180 min. A comparison of the change in activity features in the two treatment groups indicated that 5 nM DA treatment stabilized several LP activity features over the long-term relative to saline controls (Figure 5). The absolute fold-changes in LP-on phase, duty cycle and the number of spikes per burst were significantly greater in the saline relative to the DA-treatment group. On the other hand, the absolute fold-changes in LP cycle frequency, intraburst spike frequency and burst duration were not significantly different between the two treatment groups. These findings are consistent with previous population studies (Bucher et al., 2005; Goaillard et al., 2009). A positive correlation between I_A_ and I_h_ has been shown to maintain LP-on phase (Zhao and Golowasch, 2012; Krenz et al., 2013), but it is not clear if the correlation between I_A_ and I_h_ underpins the other conserved activity features and/or if DA maintains additional conductance correlations.

**Figure 6:**
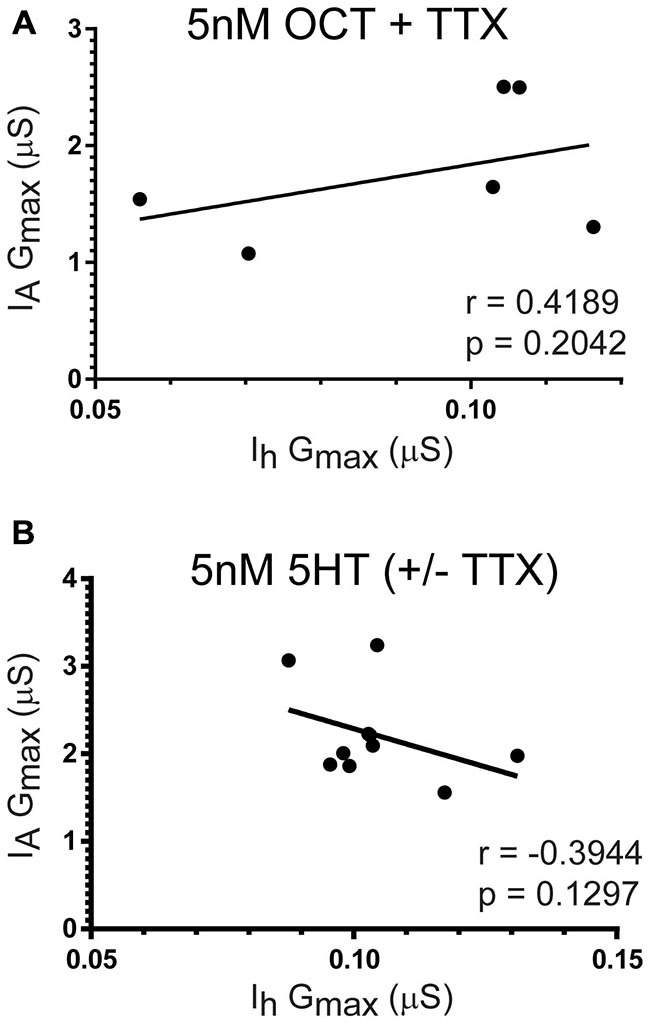
**Neither OCT nor 5HT maintained the LP I_A_:I_h_ ratio over the long-term.** Preparations were superfused with 5 nM OCT + TTX **(A)** or 5 nM 5HT (± TTX) **(B)** for 1 h, followed by a 2 h saline superfusion and TEVC to measure LP I_A_ and I_h_. LP I_A_ and I_h_ G_max_ were plotted for each individual preparation (filled circles), and the data in the scatterplots were used to compute the Pearson correlation coefficient (*r*) and associated *p*-value recorded on each plot. In addition, lines of best fit were drawn on the scatterplots.

5HT did not enable I_A_ or I_h_ plasticity or metaplasticity. Experiments on preparations superfused with 5 nM 5HT or 5 nM 5HT + TTX for 1 h followed by a 2 h saline superfusion showed that 5HT did not maintain a correlation between I_A_ and I_h_ over the long-term (Figure 6A; pooled data). OCT enabled activity-dependent regulation of LP I_A_ but not I_h_; however, 5 nM OCT + TTX did not elicit a long-term correlation (Figure 6B).

### Monoamines Elicit PD Plasticity and Metaplasticity

**Figure 7:**
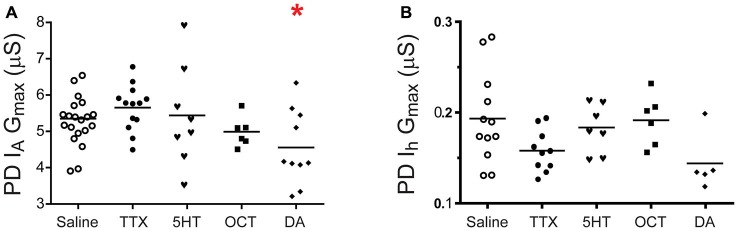
**DA, but not 5HT, OCT or a change in activity produces a persistent change in PD I_A_.** Preparations were superfused with saline, 5 nM of a given monoamine or 100 nM TTX for 1 h followed by a 2 h superfusion with saline and TEVC measures of PD I_A_ **(A)** and PD I_h_ **(B).** G_max_ are plotted; each symbol is one preparation; the lines represent means. Data in **(A)** were analyzed with one-way ANOVAs followed by Dunnett’s *post hoc* tests that compared monoamine and TTX treatment groups to the saline treatment group. Asterisks indicate significant differences between monoamine and saline treatment group; PD I_A_: *F*_(4,51)_ = 3.776, *p* = 0.0092. PD I_h_: the 5 nM DA dataset was not normally distributed and a Kruskal-Wallis test was used to compare treatment groups, *p* = 0.0503.

LP and PD express distinct sets of monoaminergic GPCRs. We examined long-term regulation of PD I_A_ and I_h_ by monoamines. Preparations were superfused for 1 h with either saline, saline containing a given monoamine or saline containing TTX; this was followed by a 2 h saline wash and TEVC to measure I_A_ and I_h_. The data suggested that only tonic nM DA could elicit I_A_ plasticity in PD and I_h_ plasticity was not observed (Figure 7). Voltage dependencies of I_A_ and I_h_ activation and inactivation were not persistently altered by any treatment.

**Figure 8:**
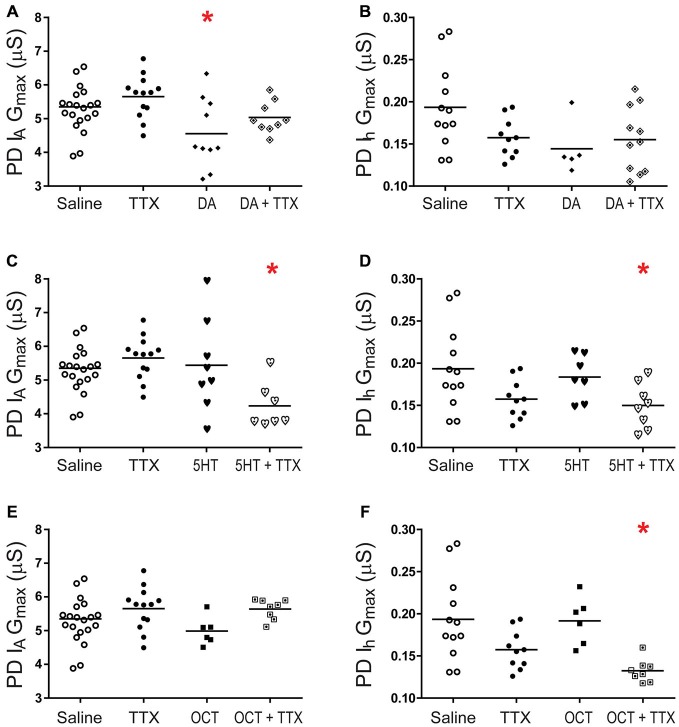
**PD metaplasticity.** Preparations were superfused with 5 nM of a given monoamine + 100 nM TTX for 1 h followed by a 2 h superfusion with saline and TEVC measures of PD I_A_ and I_h_. G_max_ are plotted; each symbol is one preparation; lines represent means. One way ANOVAs with Dunnett’s *post hoc* tests compared monoamine alone, TTX alone and monoamine + TTX treatment groups to the saline control. Asterisks indicate significantly different from control. **(A)** PD I_A_ in DA, *F*_(3,48)_ = 4.705, *p* = 0.0059;** (B)** PD I_h_ in DA: the 5 nM DA dataset was not normally distributed and a Kruskal-Wallis test was performed, *p* = 0.1203; **(C)** PD I_A_ in 5HT, *F*_(3,44)_ = 4.825, *p* = 0.0055; **(D)** PD I_h_ in 5HT *F*_(3,33)_ = 3.359, *p* = 0.0303; **(E)** PD I_A_ in OCT, *F*_(3,43)_ = 2.5433, *p* = 0.0687; **(F)** PD I_h_ in OCT, *F*_(3,32)_ = 6.125, *p* = 0.0021.

The next set of experiments investigated I_A_ and I_h_ metaplasticity. A given monoamine was co-applied with TTX for 1 h followed by a 2 h saline superfusion and TEVC to measure PD I_A_ and I_h_ (Figure 8). Interestingly, DA-elicited I_A_ plasticity was blocked by a change in activity (Figure 8A), suggesting that DA may in fact enable PD I_A_ metaplasticity such that DA permits decreases in activity to increase I_A_. PD I_A_ and I_h_ metaplasticity were observed in 5 nM 5HT + TTX (Figures 8C,D), and PD I_h_ metaplasticity was observed in 5 nM OCT + TTX (Figure 8F). Voltage dependencies of I_A_ and I_h_ were not persistently altered. The data in Figures 2, 3, 7, 8 are consistent with the idea that tonic nM concentrations of monoamines can often play a permissive role in persistent, activity-dependent regulation of ion current densities.

### RISC is Necessary for PD Ion Current Metaplasticity in 5HT but not OCT

**Figure 9:**
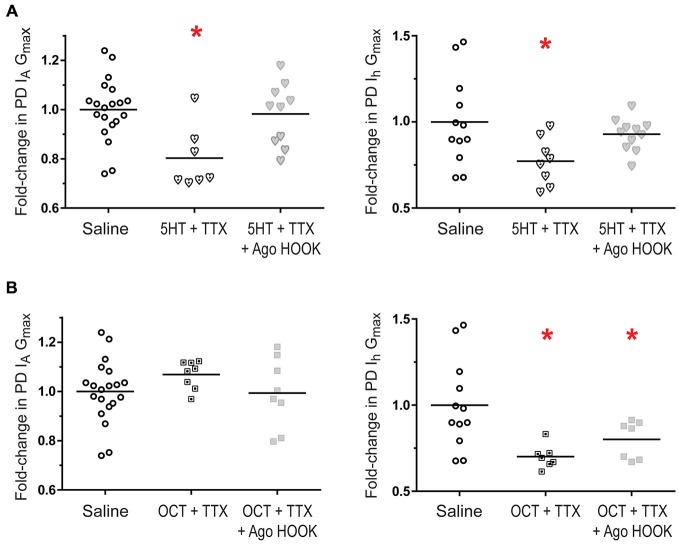
**PD metaplasticity in 5HT but not OCT requires a functional RISC.** PD neurons were injected with the Ago hook peptide to prevent RISC formation, or the mutant hook peptide as a control (Krenz et al., 2014), and were superfused with saline for 1 h. Preparations were then superfused with 5 nM 5HT + TTX **(A)** or 5 nM OCT + TTX **(B)** for 1 h followed by a 2 h saline superfusion and TEVC to measure PD I_A_ and I_h_. Saline preparations and monoamine preparations were normalized by the mean for saline preparations. Measurements for hook preparations were normalized by the means from mutant hook treatment groups. Fold-changes were plotted; each symbol is one preparation; the lines represent means. Data were analyzed with one-way ANOVAs and Dunnett’s *post hoc* tests that compared each treatment to saline. Asterisks indicate significantly different from saline. **(A)** PD I_A_ in 5HT, *F*_(2,34)_ = 6.551, *p* = 0.0039; PD I_h_ in 5HT, *F*_(2,28)_ = 3.694, *p* = 0.0079; **(B)** PD I_A_ in OCT, *F*_(2,33)_ = 1.104, *p* = 0.3434; PD I_h_ OCT, *F*_(2,23)_ = 5.982, *p* = 0.0081.

RISC was necessary for LP I_h_ metaplasticity in 5 nM DA (Krenz et al., 2014). To determine if RISC was necessary for PD I_A_ and I_h_ metaplasticity in 5HT (Figures 8B,C) or OCT (Figure 8E), the Ago hook was injected 1 h prior to superfusing the preparation for 1 h with a given monoamine + TTX. This treatment was followed by a 2 h saline wash and TEVC to measure PD I_A_ and I_h_. The data indicated that blocking RISC formation prevented PD I_A_ and I_h_ metaplasticity in 5HT (Figure 9A). On the other hand, PD I_h_ metaplasticity in OCT was RISC-independent (Figure 9B). Thus, multiple mechanisms can mediate metaplasticity in monoamines.

### Monoamines Maintain Correlations Between PD I_A_ and I_h_

**Figure 10:**
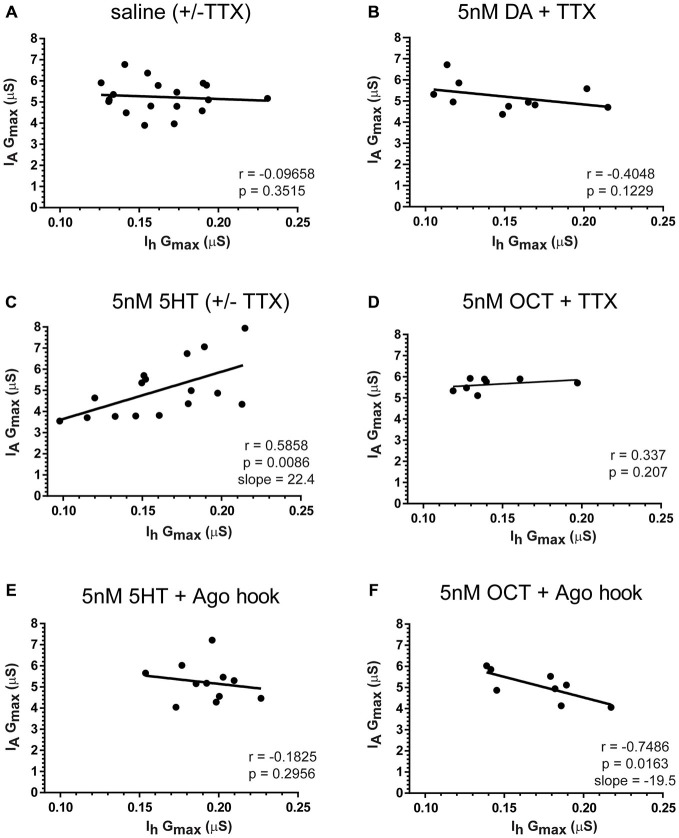
**5HT and OCT, but not DA, acted through distinct mechanisms to maintain a correlation between PD I_A_ and I_h_ over the long term.** Preparations were superfused with saline (± TTX) **(A)** or 5 nM DA + TTX **(B)** or 5 nM 5HT (± TTX) **(C)** or 5 nM OCT + TTX **(D)** for 1 h, followed by a 2 h saline superfusion and TEVC to measure LP I_A_ and I_h_. LP I_A_ and I_h_ G_max_ were plotted for each individual preparation (filled circles), and the data in the scatterplots were used to compute the Pearson correlation coefficient (*r*) and associated *p*-value that are recorded on each plot. In addition, lines of best fit were drawn on the scatter plots. Slopes are indicated for those treatment groups showing a correlation. **(E,F)** RISC mediates the positive correlation between I_A_ and I_h_ in 5HT, but masks a negative correlation in OCT. PD neurons were injected with the Ago hook peptide to prevent RISC formation and superfused with saline for 1 h. Preparations were then superfused with 5 nM 5HT + TTX **(E)** or 5 nM OCT + TTX **(F)** for 1 h followed by a 2 h saline superfusion and TEVC to measure LP I_A_ and I_h_. Scatterplots shows data for each individual (filled circles).

Each monoamine elicited a distinct form of PD metaplasticity. The data suggested that potential conductance correlations in DA and OCT would change with activity, but the correlation in 5HT may not depend on activity levels, i.e., PD I_A_ and I_h_ are similarly regulated by activity in 5HT. We therefore examined correlations in DA and OCT at a given activity level (blockade in TTX), but data from 5HT and 5HT + TTX preparations were pooled. Preparations were superfused for 1 h with saline ± TTX (Figure 10A), 5 nM DA + TTX (Figure 10B), 5 nM 5HT ± TTX (Figure 10C) or 5 nM OCT + TTX (Figure 10D), followed by a 2 h saline superfusion and TEVC to measure PD I_A_ and I_h_. The data indicated that the PD I_A_:I_h_ ratio was not maintained over the long-term in saline, DA + TTX or OCT + TTX. On the other hand, when experiments were conducted with 5HT, a positive correlation was observed, and this was disrupted by inhibiting the RNAi pathway with Ago hook injections (Figure 10E). Surprisingly, inhibiting the RNAi pathway with Ago hook injections revealed a negative correlation in OCT + TTX (Figure 10F).

## Discussion

Conductance correlations underpin neuronal activity features (Marder et al., 2015). We have been studying activity-dependent feedback mechanisms that maintain electrophysiological traits in the LP neuron, a component of the pyloric pattern generator (Marder and Bucher, 2007). LP I_A_ and I_h_ are co-regulated to maintain the timing of LP activity. When LP I_A_ G_max_ was experimentally increased or decreased, compensatory mechanisms produced corresponding changes in LP I_h_ G_max_ (MacLean et al., 2005; Rodgers et al., 2011a; Krenz et al., 2013, 2014, 2015). Tonic nM DA enabled two such mechanisms: the first was fast and acted over minutes to permit a reversible, activity-dependent regulation of LP I_h_ G_max_ that preserved a positive correlation between LP I_A_ and I_h_ and the timing of LP activity over the short-term (Krenz et al., 2013, 2015). The second mechanism was slow and acted over hours to produce persistent, activity-dependent changes in LP I_h_ G_max_ as well as an activity-independent average 25% increase in LP I_A_ G_max_ (Krenz et al., 2014). Here, we showed that the slow mechanism maintained the LP I_A_:I_h_ ratio over the long-term for a given activity state (e.g., activity blockade in TTX). This study also demonstrated that tonic nM DA stabilized the timing of LP activity, the number of spikes per burst and LP duty cycle over the long-term. Expanding our study to include additional monoamines and cell types also revealed important organizing principles for the long-term maintenance of conductance correlations by tonic nM levels of monoamines: first, every monoamine examined can act at tonic nM concentrations to enable a slow process that generates persistent activity-dependent alterations in ionic current densities. Second, persistent effects are orchestrated by multiple mechanisms even in the same cell: RISC is necessary for 5HT-enabled, but not OCT-enabled persistent activity-dependent regulation of PD I_h_. Third, the effects of a given monoamine are cell type specific: tonic nM DA preserved the LP I_A_:I_h_ ratio but not the PD I_A_:I_h_ ratio; 5HT and OCT each preserved a distinct I_A_:I_h_ correlation in PD but not LP.

### Each Monoamine Acts Through a Distinct Mechanism

The mechanism maintaining a long-term correlation between I_A_ and I_h_ appeared to vary with the monoamine. The first distinction was that tonic nM 5HT enabled activity-dependent regulation of both PD I_A_ and I_h_ G_max_, while tonic 5 nM DA enabled activity-dependent regulation of LP I_h_ G_max_ and produced a fixed, average 25% increase in LP I_A_ G_max_. This means that in 5HT, PD I_A_:I_h_ will remain fixed as PD activity varies; but in DA, LP I_A_:I_h_ will vary with activity. This was unexpected because tonic nM DA produced long-term stabilization of activity features that were strongly influenced by the I_A_:I_h_ ratio, such as LP-on phase. Since different sets of intrinsic and synaptic conductances can give rise to equivalent activity patterns (Prinz et al., 2004; Marder, 2011), it is possible that tonic nM DA confers activity-dependence on additional conductances and unique solutions based on different combinations of conductances are found for different activity states (O’Leary et al., 2014), but the same solution will be observed in each individual exhibiting a given activity state in tonic nM DA alone.

A second distinction occurs at the molecular level. DA and 5-HT confer activity-dependence through a RISC-dependent process, while OCT does not. RISCs mediate microRNA effects (Finnegan and Pasquinelli, 2013). MicroRNAs coordinate expression programs during development and in the adult (Costa-Mattioli et al., 2009; Inui et al., 2010; Goldie and Cairns, 2012; Lee and Vasudevan, 2013; Ma et al., 2013; Sun et al., 2013). One microRNA co-regulates the translation of tens to hundreds of mRNAs, sometimes in an activity- and/or G-protein coupled receptor-dependent fashion (Costa-Mattioli et al., 2009; Impey et al., 2010; Cohen et al., 2011; Saba et al., 2012; Eacker et al., 2013). Each monoamine could maintain a conductance correlation by acting through a given microRNA to co-regulate the translation of Kv4 channels and hyperpolarization activated cyclic nucleotide gated (HCN) channels that mediate I_h_. Correlations in ion channel transcript numbers have been observed in a number of cell types (Schulz et al., 2006, 2007; Tobin et al., 2009), and they can be maintained in an activity-dependent manner (Temporal et al., 2014), suggesting that microRNA may also co-regulate the translation of a transcription factor that acts at the promoters of both Kv4 and HCN channel genes (Bredy et al., 2011; Tan et al., 2012). Alternatively, some microRNAs control transcription by directly binding promoters (Zhang et al., 2014). The slow RISC-independent mechanism enabled by OCT could also act at the level of transcription and/or translation.

### Compartment Specific Controllers?

A homeostat uses negative feedback to maintain a target (e.g., Ca^2+^) at a given set point (e.g., 100 nM) by employing a sensor (e.g., calmodulin) that measures deviations from the set point and then generates a precise error signal (e.g., graded alteration in the activity of the Ca^2+^-calmodulin dependent phosphatase, calcineurin) that then feeds back onto the system to produce a change (e.g., graded calcineurin-induced alteration in ion channel surface expression) that then returns the target to its set point (Davis, 2006). A recent computational model of a homeostat (a.k.a. controller) with a Ca^2+^ target was able to successfully maintain conductance correlations and activity features by regulating ion channel transcription in an activity-dependent fashion (O’Leary et al., 2014). In this model, average Ca^2+^ concentration depended upon neuronal activity, and a Ca^2+^-dependent process drove the transcription of ion channel genes, e.g., A and B. The model assumed that functional protein expression linearly correlated with mRNA concentration, which is not always the case. Nevertheless, given this assumption, the model demonstrated that the ratio of “regulation rate constants” for A and B defined the A:B conductance ratio (O’Leary et al., 2013, 2014). The regulation rate constant for a given gene reflected the Ca^2+^-dependent rates of mRNA synthesis and degradation. The model used one controller but did not preclude the use of multiple parallel controllers with unique targets in one cell. Here, we interpret our data in the context of controllers, but other interpretations that do not require homeostats and set points are also possible.

Do tonic nM concentrations of monoamines activate controllers or simply influence expression rate constants? Our studies on the fast DA-enabled mechanism showed that tonic 5 nM DA permitted Ca^2+^-calmodulin-dependent calcineurin to regulate LP I_h_ G_max_ (Krenz et al., 2015), suggesting that DA allowed the error signal to feed back onto the system, thereby closing an open activity-dependent feedback loop. Furthermore, there was no correlation in the absence of tonic nM DA. These data suggest that tonic low-level concentrations of monoamines can activate controllers (or modular feedback within a master controller) by closing open loops, as well as influence the expression rate constants that define correlations. Peptide modulators may have similar functions (Khorkova and Golowasch, 2007).

How are distinct monoamine-enabled, activity-dependent feedback loops organized within a cell? OCT alone produced no correlation between PD I_A_ and I_h_ G_max_, though it did enable activity-dependent regulation of PD I_h_. However, when RISC was blocked OCT produced a negative correlation between PD I_A_ and I_h_ G_max_. Interestingly, 5HT maintained a RISC-dependent positive correlation. Do individual monoamines influence specific controllers in distinct compartments or is there one centralized controller that is influenced in a collective manner by all elements of modulatory tone? In the first case distinct targets are maintained; e.g., distinct Ca^2+^ pools, or Ca^2+^ vs. cAMP, etc. In the second situation, a single target is maintained. Each case is considered here.

Several compartment-specific controllers could exist in one cell. The space-clamped area from which measures of I_A_ and I_h_ derive comprises multiple subcellular compartments. For the sake of argument, assume that two distinct sectors exist within the space-clamped region: the somatodendritic compartment and spike initiation zone (siz). If 5HT and OCT were co-applied and 5HT maintained a strong positive correlation in the somatodendritic compartment but no correlation at the siz, and the opposite were true for OCT, then the correlation in the larger compartment could mask the correlation in the smaller compartment, for example. Studies show that mRNA, microRNA, monoamine receptors, second messengers, signaling components and their targets can all be highly localized (Steward, 1997; Zaccolo and Pozzan, 2002; Cooper, 2005; Few et al., 2007; Schratt, 2009; Oginsky et al., 2010; Lin et al., 2011; Swanger and Bassell, 2011; Kindler and Kreienkamp, 2012; Dacher et al., 2013). Thus, the 5-HT pathway could regulate transcription and translation by acting on microRNA and mRNA localized to dendrites while the OCT pathway could act at the siz to control local ion channel surface expression. The idea of compartment specific controllers could account for opposing conductance and mRNA correlations (Ransdell et al., 2012) and correlations at the transcript but not conductance levels (Zhao and Golowasch, 2012). However, it should be noted that opposing homeostats can act in a hierarchical fashion (Bergquist et al., 2010). In this event, the 5HT and OCT controllers would operate in a mutually exclusive manner.

In the second case, a single master controller regulates the entire population of Kv4 and HCN channels in the space-clamped compartment. Here, processes enabled by 5HT and OCT would influence multiple expression time constants for each channel, and thereby shape one I_A_:I_h_ conductance correlation based on their relative strengths. In this model, all monoamine-elicited processes would maintain the same target set point, but it is not clear how the different monoamine pathways would be integrated into the master controller because so little is known about their transduction cascades. Presumably, a master controller could incorporate multiple sensors and/or error signals to create a variety of feedforward and feedback loops (Davis, 2006). Here, it is worth repeating that tonic 5 nM DA actuates two PKA-mediated mechanisms that function over distinct time courses to maintain a positive correlation between LP I_A_ and I_h_ G_max_ by endowing I_h_ with activity dependence (Krenz et al., 2014, 2015). Both fast and slow DA-enabled activity-dependent regulation of LP I_h_ G_max_ could be mediated by one controller that uses a single sensor and error signal to generate divergent downstream effects.

### The Role of Tonic Modulators

The function of tonic modulators may differ from phasic modulation in that the former stabilizes while the latter alters neuronal activity. Studies show that tonic DA can act on unmyelinated axons to improve the temporal fidelity of spike propagation and elicit ectopic peripheral spiking in the absence of centrally generated bursts; these actions appear to be graded over a 100 pM−1.0 μM concentration range (Bucher et al., 2003; Ballo et al., 2010, 2012). Tonic DA can also act in the neuropil to generate persistent alterations in G_max_ amplitudes that stabilize neuronal output, but these responses may not be dose-dependent. DA-induced the same persistent ~25% increase in LP I_A_ G_max_ over a concentration range that spanned 4 logs (500 pM–5 μM) and no persistent effect was observed at 50 pM DA (Rodgers et al., 2011b, 2013). Thus, if high affinity D1Rs regulate LP I_A_ G_max_ in a dose-dependent fashion, they do so over a very limited range of DA concentrations (50–500 pM). Likewise, LP I_h_ G_max_ metaplasticity in DA is not graded in the nM-μM range: 5 nM DA + TTX and 5 μM DA + TTX evoked the same persistent ~50% increase in LP I_h_ G_max_ (Rodgers et al., 2011a; Krenz et al., 2014). Perhaps this was because the change in activity determined the extent of the alteration in G_max_, and tonic DA only permitted that regulation to occur. Monoamines are tonically present at nM concentrations (~1–30) in the mammalian brain (Fitzgerald, 2009; Owesson-White et al., 2012). The same is probably true for the STG which receives volume and neurohormonal monoamine transmissions. Together the data suggest that tonic monoamine levels may normally saturate high affinity receptors to constitutively activate controllers. Neuromodulators and their receptors are present and functional during the embryonic development of STGs circuits (Fenelon et al., 1999; Kilman et al., 1999; Le Feuvre et al., 2001; Pulver et al., 2003; Cape et al., 2008; Rehm et al., 2008a,b). Conceivably, low level modulatory tone could act throughout development and lifetimes to persistently engage controllers that specify cell types (Das and Bhattacharyya, 2014). In circuits/systems where tonic levels periodically fluctuate, monoamines could also engage controllers to maintain distinct states (Monti, 2011; Puig and Gener, 2015).

## Funding

This work was supported by NSF IOS-1456971.

## Conflict of Interest Statement

The authors declare that the research was conducted in the absence of any commercial or financial relationships that could be construed as a potential conflict of interest.
